# Boundedly rational bidding decision for land auctions during the transformation of real estate markets

**DOI:** 10.1038/s41598-023-41993-7

**Published:** 2023-09-09

**Authors:** Rujin Liao, Jing Zhang, Ruwen Tan, Yilin Wu, Minjiu Yu

**Affiliations:** College of Architecture and Environment, Sichuan University, No.24 South Section 1, Yihuan Road, Chengdu, 610065 China

**Keywords:** Human behaviour, Civil engineering

## Abstract

Real estate developers in China exhibit different boundedly rational behaviors when bidding in different land auction formats, resulting in deviation from fully rational bidding, and the transformation of China’s real estate industry has intensified this deviation. To address this problem, this study establishes a reference utility model and a subjective decision probability model under the framework of incomplete information game to characterize developers’ utility distortion and winning probability distortion in open-bid auctions and sealed-bid auctions for land, respectively. The results show that the deviation between bounded rational bidding and fully rational bidding increases as developer competition intensifies. The equilibrium bidding of cost-disadvantaged developers in the boundedly rational model deviates less from the standard model. Moreover, in sealed-bid auctions, the "hiding degree" of cost-advantaged developers is greater, showing size effects, while the bids in open-bid auctions are more complex and affected by market conditions and developers’ risk preferences. Thus, this study characterizes boundedly rational bidding in land auctions and interprets the deviation from fully rational bidding, which can provide a more real basis for land auction mechanism design.

## Introduction

China’s land resources have long been granted mainly through bid invitation^1^ (sealed-bid auction), auction^2^ (English auction), and listing (ascending open-bid auction in two related stages, lasting for a period of time)^3^. Real estate developers in China have to acquire land through auction before construction and operation activities. Land auction bidding is clearly crucial for developers.

Traditional auction theory usually assumes that decision-makers are fully rational^4–6^; that is, all decision-makers are able to accurately obtain all the relevant information and make the optimal decision and believe that others behave likewise. However, in practice, due to differences in information acquisition^7^, decision-making experience^8^, intelligence^9^, cognitive level^10^, emotional control^11^, etc., and irrational behavior characteristics, decision results often deviate from rational predictions^12^. In particular, due to the large capital investment and long payback period of real estate^13^, developers’ bidding in land auctions is more susceptible to bounded rationality. Specifically, on the one hand, in open-bid auctions (i.e., auction and listing), developers’ values are gradually revealed through rounds of open bidding. Therefore, developers’ utility from auctioned land will be distorted by competitors’ bids and market conditions, thus distorting their bidding strategies. On the other hand, in sealed-bid auctions (i.e., bid invitation, usually for land with specific uses), since competitors’ bids are unobservable, developers’ bids are closer to their true values. However, developers’ estimated winning probability might be distorted by overconfidence and the gambler effect^14^. In brief, developers’ irrationality about utility and winning probability in different auction formats deviates their bids from rational predictions. Therefore, it is significant to characterize boundedly rational bidding in land auctions.

In addition, the changes in China’s real estate market also create new difficulties in land auction bidding. During the rapid growth of Chinese real estate in the past decade, the competition between developers remains incomplete, and as a result, the deviation of bidding strategies under the fully rational and boundedly rational models is relatively small. However, different from the past when development could be profitable^3^, China’s real estate market is undergoing transformation. Since 2022, despite the relaxation of mortgage policies in many places, China’s land transaction price and cumulative sales of commercial housing still fell by 46.3% and 28.9%, respectively, compared with the previous year (stats.gov.cn). In the case of declining revenue, the loss caused by poor bidding decision will further threaten developers’ viability^15^. As a result, it is imperative to study land auction bidding under the assumption of bounded rationality in the context of the transformation of China’s real estate industry.

On this basis, this study establishes the reference utility model and the subjective decision probability model under the framework of the incomplete information game to describe the two kinds of boundedly rational behaviors of developers (i.e., distortions of utility and winning probability) in the open-bid auction and sealed-bid auction for land, respectively. By solving the Bayesian-Nash equilibrium, the optimal bidding strategies of the two models are obtained and compared with fully rational bidding. Furthermore, the two boundedly rational bidding strategies are also comparatively analyzed, and the corresponding applicable conditions of the two models are obtained.

The contributions are as follows. Firstly, the research findings of behavioral economics are creatively introduced into the land auction bidding in this study, which enriches research on bounded rationality in land auctions. Secondly, two boundedly rational bidding models are proposed, providing decision support for developers in different formats of land auctions. Thirdly, the deviation between boundedly rational bidding and fully rational bidding is depicted and interpreted by accurately characterizing two boundedly rational behaviors using models, which provides a more real basis for land auction mechanism design. Moreover, this study focuses on land auction bidding during the transformation of China’s real estate industry, which contributes to the healthy development and virtuous cycle of China’s real estate industry, and provides a reference for the development of the real estate industry in developing countries.

## Literature review

The literature review mainly focuses on the bidding decisions in land auctions and the bounded rational behavior of bidders in auctions.

### Bidding decisions in land auctions

Land auctions are a prominent issue in China, featuring the particular characteristics of public ownership and price limits. Most previous studies are based on the assumption of full rationality. On the one hand, some studies focus on equilibrium bidding in auctions. For example, Yang and Peng^16^ analyze bidding strategies in a random limit auction using non-cooperative game theory and obtain Nash equilibrium bidding strategies in differential form. In a study of land auctions in eastern Germany, a structural estimation approach is applied to determine reserve prices and to compare the resulting expected revenues with actual revenues^17^. On the other hand, some studies investigate land auction mechanism design, including bidding strategies. Wu et al.^4^ derive the equilibrium strategies of developers through symmetric Nash equilibrium with developers modeled as rational agents and presents applicability conditions for different land auction mechanisms. For high housing prices, Zhang and Wang et al.^5^ design an auction mechanism that includes land prices and housing prices using multi-attribute auction, introducing housing price control into state-owned land auctions. On this basis, Cai and Guo^6^ propose a two-stage auction mechanism of "limiting land price and competing promised house price" and use expected utility theory to derive the equilibrium bidding decisions of land bidders under the condition of full rationality. Considering the complementarity between construction land and the quota of construction land, Deng et al.^18^ establish a two-stage sequential auction model to study the pricing mechanism of the quota.

In these studies, a classical model of a non-cooperative game is established under the assumption that bidders are risk neutral, and Bayesian-Nash equilibrium is obtained. However, the boundedly rational behaviors of bidders and resulting deviation in bidding^12^ have rarely been discussed. Different from previous studies, this study characterizes two kinds of boundedly rational behaviors in open-bid auctions and sealed-bid auctions, i.e., the utility distortion and the winning probability distortion. A brief comparison is in Table 1.

**Table 1:** Bidding decisions in land auctions.

Reference	Assumption framework	Research focus	Market conditions	Auction formats
Yang and Peng^16^	Fully rational	Equilibrium bidding	–	First-price sealed-bid auction and English auction
Marlene et al.^17^	Fully rational	Equilibrium bidding	–	First-price sealed-bid auction
Wu et al.^4^	Fully rational	Equilibrium bidding and mechanism design	Housing price control	First-price sealed-bid auction
Zhang and Wang^5^	Fully rational	Equilibrium bidding and mechanism design	–	First-price sealed-bid auction
Cai and Guo^6^	Fully rational	Equilibrium bidding and mechanism design	Limiting land price	First-price sealed-bid auction
Deng et al.^18^	Fully rational	Equilibrium bidding and mechanism design	–	First-price sealed-bid auction and second-price sealed-bid auction
Peng and Liu^19^	Bounded rational	Equilibrium bidding	–	First-price sealed-bid auction
This study	Bounded rational	Equilibrium bidding	Market transformation	First-price sealed-bid auction

### The boundedly rational behavior of bidders in auctions

In recent years, bounded rationality in auctions has attracted increasing attention. For instance, Adomavicius et al.^20^ discuss the effect of decision complexity on the outcome of combinatorial auctions using the results of an experiment conducted by college students and find that increasing competition forces bidders to find "loopholes" in the auction to obtain a surplus. Qian^21^ considers the decisions of boundedly rational bidders at different levels of thinking in a reverse auction with first-price sealed bids and finds that any bidder with more than two levels of thinking should bid according to the strategy of the second-level bidder to achieve the highest expected profit. Gao^22,23^ constructed a nonlinear function to portray bidders' regret psychology and solved the equilibrium strategy under bidder regret psychology behavior. Peng and Liu^19^ studied decision-makers' multiple auction decisions in one decision cycle and considered the influence of bidders' risk attitudes and preferences on their bid prices using accumulative prospect theory.

It has been proven that there is a deviation between the bidding decision in reality and the equilibrium bidding under the full rationality assumption. However, few studies have described the boundedly rational behavior of bidders in land auctions using mathematical models, nor have they considered the differences between different auction formats. In particular, this study focuses on two different boundedly rational behaviors of bidders in open-bid auctions (i.e., auction and listing) and sealed-bid auctions (i.e., bid invitation) in China and depicts the deviations from fully rational bidding by modeling. Moreover, since the transformation of the real estate industry is a general trend following the experience of real estate in developed countries^24^, this study also considers the challenges brought by the transformation of China's real estate market to land auction bidding. A brief comparison is in Table 2.

**Table 2:** The bounded rationality of bidders in auctions.

Reference	Distorted subjective conditions	Method
Adomavicius et al.^20^	Complexity	Experimental design
Qian^21^	Expected profits	Mathematical model
Gao^22,23^	Utility distortion	Mathematical model
Peng and Liu^19^	Cumulative prospect theory	Mathematical model
Shachat and Tan^25^	Probabilistic distortion	Mathematical model and experimental design
This study	Utility distortion and probability distortion	Mathematical model

## Problem description

Based on the above research background, similar to existing studies^1,6,20^, we propose the following scenario: in a land auction for a land parcel to be auctioned (hereafter the target land), the grantor is the government (hereafter the grantor), and there are $n$ potential bidders (hereafter bidder $i$), $n \ge 2$. The rules of the auction are that $n$ bidders submit sealed bids, and then the grantor selects the highest bid from all the bids and grants the target land at that price. The value of the target land for bidder $i$ is ${v}_{i}$, which is the bidder's private information. ${v}_{i}$ is an abstraction that includes the bidder’s development costs for the target land and the revenue from the sale of the house to consumers. The value, ${v}_{i}$, also indicates a developer’s profitability, e.g., technical level, the brand and the special relationship with the owner, etc., which will also affect the developer's expected return on the auctioned land. The sealed bid for the target land is $x_{i} ,\;\;1,2, \ldots n.$ For convenience, we rank the bids in descending order, i.e., ${X}_{1}=\mathrm{max}\left\{{x}_{1}, {x}_{2},\dots {x}_{n}\right\}$.

Bidders are boundedly rational in their bidding decisions with risk preferences and reference effects. On the one hand, bidders subjectively distort the utility generated by the target land^26,27^. On the other hand, bidders may also subjectively distort the probability of winning^25,28^.

This study focuses on two different boundedly rational behaviors of bidders in open-bid auctions (i.e., auction and listing) and sealed-bid auctions (i.e., bid invitation) in China. Open-bid auctions reflect perfect competition in which the highest bidder wins. Sealed-bid auctions are usually for land with specific uses, and competitors’ bids are unobservable. Therefore, a standard model under the full rationality assumption and two bidding models under the bounded rationality assumption (i.e., reference utility model and subjective decision probability model) are developed. The difference between fully rational and boundedly rational decisions is analyzed theoretically.

### Assumptions

1. The models in this study are established under the framework of the symmetric independent private value (SIPV) model. The value of the auctioned land of bidder $i$ is ${v}_{i}$. ${v}_{i}$ is the bidder's private information, and its distribution independently follows the interval $\left[\underline{v},\overline{v }\right]$. The distribution function of $F\left({v}_{i}\right)$ and its density function is $f\left({v}_{i}\right)$. The distribution function and the number of participants in the auction are public information.
2. In the fully rational model, bidders are assumed to be fully rational. We consider the ideal condition that when the developer wins the auction, the developer's surplus is its value minus its bid price; otherwise, its surplus is $0$. Then the developer's surplus ${\pi }_{i}$ is given as follows:
  1 $$\begin{array}{c}{\pi }_{i}=\left\{\begin{array}{ll}{v}_{i}-{x}_{i} & \quad if\, {x}_{i}>\underset{j\ne i}{\mathit{max}}\,{x}_{j},i=\mathrm{1,2}\dots ,n\\ 0 & \quad or\end{array}\right.\end{array}$$
  In the boundedly rational models, it is assumed that all bidders treat the bidding decision in land auctions with bounded rationality. Referring to the behavioral finance research on decision-makers, we will consider that realistic people have the following irrational behaviors when making decisions^7,27,28^:
  i. Reference effects: boundedly rational people tend to evaluate gains and losses based on the reference point.
  ii. Loss aversion: boundedly rational people are more sensitive to losses than to gains.
  iii. Diminishing sensitivity: based on the reference point, the bidder's marginal benefit for both gains and losses is decreasing.
  iv. The gambler's effect: boundedly rational people tend to overestimate the impact of low-probability events.
  v. Overconfidence: boundedly rational people tend to overestimate the impact of high-probability events.
  Irrational behaviors i-iii describe realistic people’s perception of the utility; that is, they evaluate the actual utility subjectively and convert it into subjective utility. iv-v depict bidders' subjective distortion of the objective probability of winning the bid, and the bidding decisions are made based on the subjective decision probability.
  To characterize two different kinds of bounded rationality in land auctions from the perspective of behavioral economics, this study establishes two modes of bounded rationality assumption corresponding to open-bid and sealed-bid auctions:
3. In the open-bid auctions, bidders’ utility from the target land might be affected by competitors’ bids as rounds of open bidding proceeds, thus changing their own bidding strategies. Therefore, it is assumed that bidders in the land auction view the probability of winning rationally but are irrational about the utility brought by the target land, and bidders exhibit reference dependence, loss aversion, and decreased sensitivity. This is summarized as bounded rationality assumption I.
4. In sealed-bid auctions, since competitors’ bids are unobservable, bidders remain firm in their value and utility of land and will not change easily. However, opacity of the bidding tend to induce subjective distortion of winning probability. Therefore, it is assumed that bidders view the utility brought by the target land rationally but distort the winning probability, and bidders show the characteristics of the gambler's effect and overconfidence. This is summarized as the bounded rationality assumption II.

## Models

In this section, we formulate the standard model under the assumption of fully rational bidders, the reference utility model (RUM) under bounded rationality assumption I, and the subjective decision probability model (SDPM) under the bounded rationality assumption II.

### Standard model

In the standard model, bidders are assumed to be fully rational, and decisions are made according to expected utility theory.

Each of the $n$ bidders determines his bid according to his value of the target land. Thus, let ${x}_{i}=\phi \left({v}_{i}\right)$ be the equilibrium bidding strategy function of bidder $i$. In the symmetric independent private value framework, ${x}_{i}=\phi \left({v}_{i}\right)$ exists and is strictly continuous, increasing, and differentiable in $\left[\underline{v},\overline{v }\right]$^29^. Then, ${v}_{i}={\phi }^{-1}\left({x}_{i}\right)$ exists and has the same property as $\phi \left({v}_{i}\right)$. For simplicity, we introduce $G$ to denote the cumulative distribution function of ${X}_{1}$ (${X}_{1}$ is the highest bid among all bids), and it is clear that $G={F}^{n-1}$ . Then, the expected utility of bidder $i$ for the target land is

2 $$\begin{array}{c}E({x}_{i})=G\left({\phi }^{-1}\left({x}_{i}\right)\right)\times {\pi }_{i}=G\left({\phi }^{-1}\left({x}_{i}\right)\right)\times \left({v}_{i}-{x}_{i}\right)\end{array}$$

$G\left({\phi }^{-1}\left({x}_{i}\right)\right)$ means that $\mathrm{Pr}[{x}_{i}>\underset{j\ne i}{\mathrm{max}}\,{x}_{j}]$. ${v}_{i}-{x}_{i}$ is the return on winning the bid. All bidders maximize their expected utility.

#### **Proposition 1**

*Under the assumption of full rationality, bidders* $i$ *'s optimal bid strategy is*

3 $$\begin{array}{c}{x}_{i}^{*}=\phi ({v}_{i})={v}_{i}-{\int }_{\underline{v}}^{{v}_{i}}{\left(\frac{F\left(t\right)}{F\left({v}_{i}\right)}\right)}^{n-1}dt , \,{x}_{i}^{*}\epsilon \left[\underline{v},\overline{v }\right]\end{array}$$

*The proof is in* Appendix A.

### Bounded rationality decision model

This section assumes that bidders conform to assumptions I and II of bounded rationality in making land auction bidding decisions. The bounded rationality decision model in this section is divided into two cases to discuss the impact of the boundedly rationality behavior of decision-makers on bidding decisions: Firstly, bidders will make a subjective evaluation of the utility generated by the target land, but the probability of winning the bid is a rational attitude, forming the RUM. Secondly, bidders will distort their probability of winning the bid, and the utility of the target land to them is a rational attitude, forming the SDPM.

#### Reference utility model

The reference utility model is still based on the SIPV framework, and only the utility bounded rationality of bidders is considered. In the study of decision-makers' behavior in behavioral finance, it is found that decision-makers distort the utility of commodities. For example, Davis et al.^26^ consider the effect of bidders' expected regret on utility. Li^27^ argues that people's decision preferences are determined by the increment of wealth rather than the total amount of wealth and that the increment of wealth is judged to be dependent on a reference point, suggesting that decision-makers do not rationally view utility. In the past 30 years of research, scholars have confirmed from various perspectives that human decision-making behavior is highly influenced by reference points. Prospect theory finds that human behavior depends not only on the increase or decrease in surplus but also on the change relative to a reference point. Barberis and Xiong^30^ propose that the importance of reference points in auctions under the disposition effect lies in the resistance that bidders experience in their decision to sell at a loss or to hold at a profit. In Baillon’s studies^31^, the reference points that people use in risky decisions are considered to be empirically determined. Based on these studies, this study concludes from interviewing experts that real estate developers will always compare their payoff to the industry benchmark rate of return. It can reflect the development state of the industry and is public reference information for bidders. The reference point for bidders in land auctions is defined as

4 $$\begin{array}{c}{r}_{0}=\frac{{v}_{i}}{1+\lambda }\end{array}$$

In Eq. (4), ${r}_{0}$ denotes bidder $i$’s reference point for the decision to bid on the target land, ${v}_{i}$ denotes bidder $i$'s value of the target land, and $\lambda$ denotes the benchmark rate of return for the real estate industry.

In addition to reference dependence, people's behavior is also characterized by loss aversion and decreasing sensitivity, i.e., for a given amount, bidders prefer to avoid losses rather than pursue gains. Additionally, the marginal utility of bidders decreases relative to the reference point.

Based on the above analysis, the subjective utility function of the bidder for the land is established.

5 $$\begin{array}{c}{\widetilde{\pi }}_{i}=\left\{\begin{array}{ll}{-\upeta \left({x}_{i}-{r}_{0}\right)}^{\beta }, & \quad if \;{x}_{i}\ge {r}_{0}\\ {\left({r}_{0}-{x}_{i}\right)}^{\alpha }, & \quad if \;{x}_{i}<{r}_{0}\end{array}\right.,\eta >1,\alpha ,\beta \in (\mathrm{0,1})\end{array}$$

**Figure 1:**
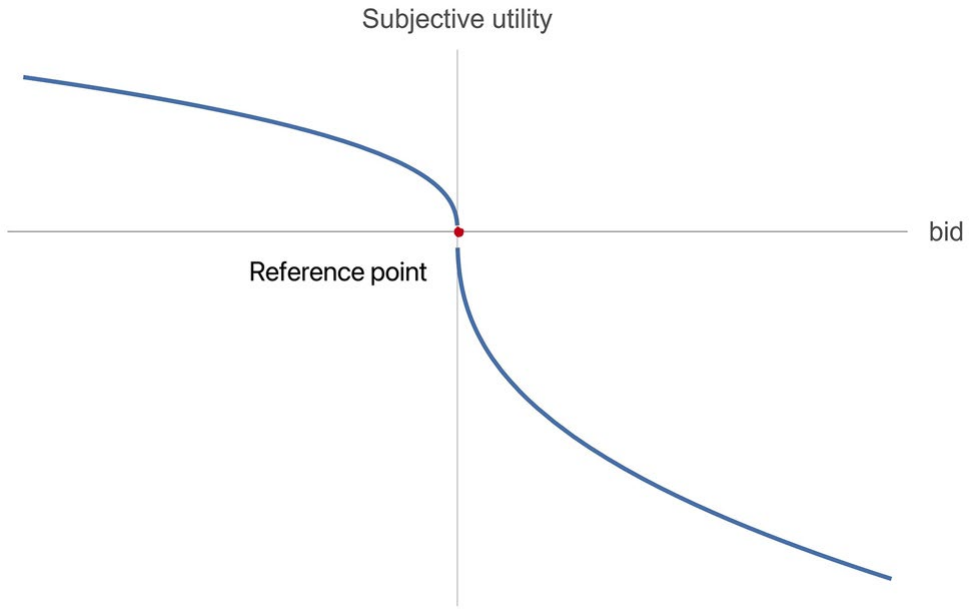
The shape of the function ${\widetilde{\pi }}_{i}$.

In Eq. (5), ${\widetilde{\pi }}_{i}$ denotes bidder $i$'s subjective value utility function for the target land, and ${x}_{i}$ represents bidder $i$'s bid for the target land, which depends on the bidder's value and bidding strategy. ${r}_{0}$ represents bidder $i$'s reference point price of the land; see Eq. (4). $\alpha ,\beta \in (\mathrm{0,1})$ is set to conform to the marginal effect, that is, to conform to $\frac{{\partial }^{2}\left({\widetilde{\pi }}_{i}\right)}{\partial {{x}_{i}}^{2}}<0$ when ${x}_{i}\ge {r}_{0}$ and $\frac{{\partial }^{2}\left({\widetilde{\pi }}_{i}\right)}{\partial {{x}_{i}}^{2}}>0$ when ${x}_{i}<{r}_{0}$ . $\alpha$ and $\beta$ denote the bidder's attitude toward gain and loss, respectively. The greater $\alpha$(or $\beta$) is, the greater the risk appetite (or the degree of risk aversion)^7^. The distribution relationship is shown in Fig. 1.

Define ${x}_{i}=\varphi \left({v}_{i}\right)$ as the bidding strategy function of the subjective value of bidder $i$. The expected utility function of bidder $i$ under the RUM is as follows.

6 $$\begin{array}{c}{E}_{\left(+\right)}\left({x}_{i}\right)=G\left({\varphi }^{-1}\left({x}_{i}\right)\right)*{\left({r}_{0}-{x}_{i}\right)}^{\alpha }\end{array}$$

7 $$\begin{array}{c}{E}_{\left(-\right)}\left({x}_{i}\right)=G\left({\mathrm{\varphi }}^{-1}\left({\mathrm{x}}_{\mathrm{i}}\right)\right)\left({-\eta \left({x}_{i}-{r}_{0}\right)}^{\beta }\right)\end{array}$$

${E}_{\left(+\right)}\left({x}_{i}\right)$ and ${E}_{\left(-\right)}\left({x}_{i}\right)$ denote the subjective expected utility of the bidder compared to the expected gain and loss at the reference point, respectively. $G\left({\varphi }^{-1}\left({x}_{i}\right)\right)$ is bidder $i$’s objective probability of winning the bid. The goal of the bidders is to maximize expected utility, which is max{${E}_{\left(+\right)}\left({x}_{i}\right), {E}_{\left(-\right)}\left({x}_{i}\right)$}.

##### **Proposition 2**

*The optimal bid strategy for a boundedly rational bidder with utility irrationality is*

8 $$\begin{array}{c}{{x}_{i}}^{*}=\frac{1}{\left(1+\lambda \right)\alpha }{\int }_{\underline{v}}^{{v}_{i}}\frac{{tG\left(t\right)}^{-1+\frac{1}{\alpha }}g\left(t\right)}{{G\left({v}_{i}\right)}^{1/\alpha }}dt, {x}_{i}^{*}\epsilon \left[\underline{v},\overline{v }\right]\end{array}$$

*The proof is in* Appendix A.

#### Subjective decision probability model

The subjective decision probability model is still based on the SIPV framework, and only the winning probability bounded rationality of bidders is considered. In sealed-bid auctions, bidders will distort the winning probability. As mentioned above, bidders have irrational subjective judgments about their probability of winning and believe that their competitors behave likewise. For example, in most cases^7,25^, small developers will also actively participate in auctions because they subjectively amplify their belief that they will win, and large developers tend to overestimate their competitors' belief of winning. In Birnbaum& Navarrete's^32^ studies, it is also shown that most phenomena can be explained by a simplification of the weight function compared to a nonlinear representation of the benefit function, and in their model, no distortion of the benefit function is required to explain these boundedly rational economic phenomena. Therefore, this subsection follows the bounded rationality assumption II that bidders in the sealed-bid auction will distort their winning probability.

Distortions of probabilities are not uncommon, e.g., Shachat and Tan^25^ used a two-parameter function to distort the subjective conditional probability of the auctioneer, and Birnbaum and Bahra^28^ used a binary gambling model to consider the probability distortion function for gamblers in different situations. This study follows the binary gambling model proposed by Birnbaum and Bahra to form a distortion function of the bidders' probability of winning.

9 $$\begin{array}{c}\omega \left({G}_{i}\right)=\frac{{{G}_{i}}^{\xi }}{{{G}_{i}}^{\xi }+{\left(1-{G}_{i}\right)}^{\xi }}\end{array}$$

**Figure 2:**
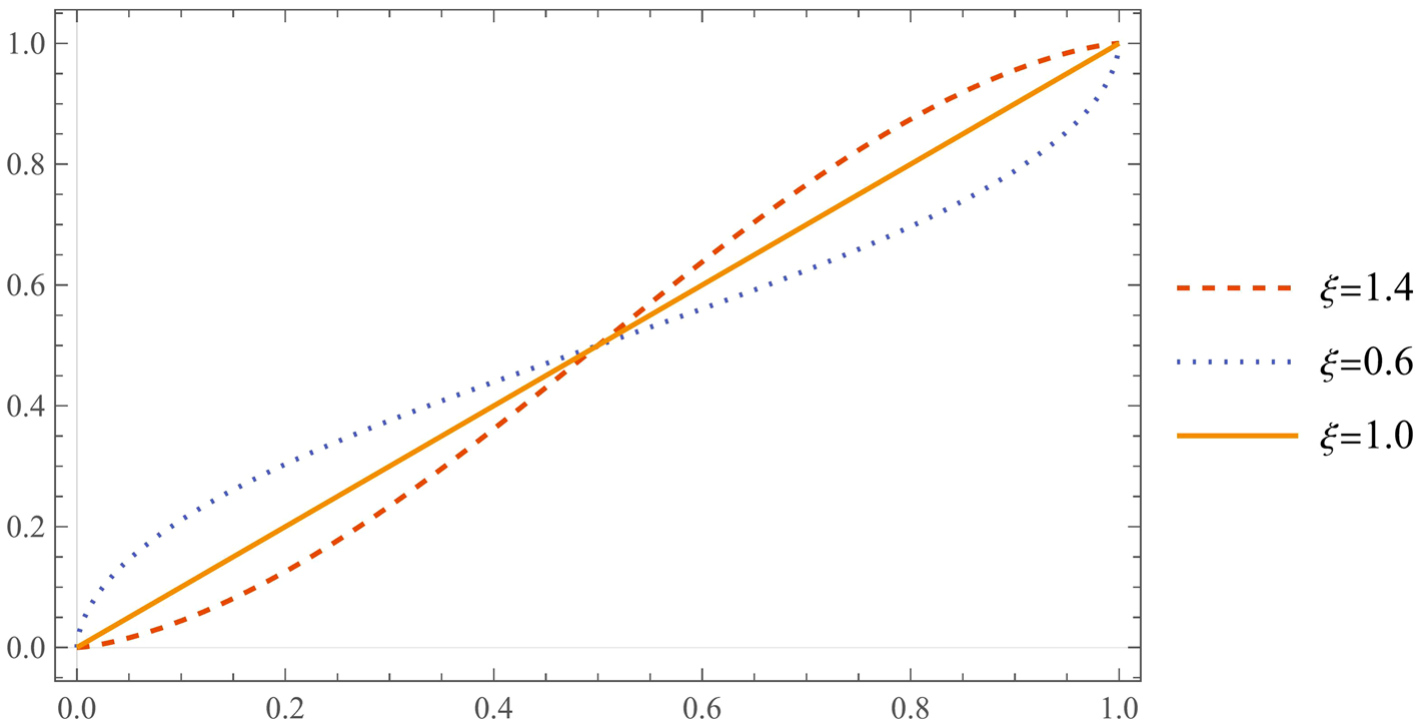
S-shaped $\omega \left({G}_{i}\right)$ when $\xi$ differs.

In Eq. (9), ${G}_{i}$ denotes the probability that a rational bidder will win the target land, and $\xi$ is the attitude coefficient of the bidder's decision-weighting attitude. When $\xi >1$, this indicates that the bidder is overconfident and irrational; when $\xi <1$, this indicates that the bidder is irrational with the gambler effect, and when $\xi =1$, this indicates that the bidders are rational. $\omega \left({G}_{i}\right)$ denotes the subjective decision probability distorted by the bidders, which is the inverse S description of the bidders' probability of winning, as Fig. 2 shows. In behavioral economics, the estimation of the winning probability is often set to follow the probability model under the condition of $\xi =0.61$. As shown in Fig. 2, the subjective winning probability of developers with lower values (i.e., cost-disadvantaged developers) is higher than the winning probability under full rationality, while the subjective winning probability of developers with higher values (i.e., cost-advantaged developers) is lower than the winning probability under full rationality.

In this setting. Define ${x}_{i}=\psi \left({v}_{i}\right)$ as the bidding strategy function of the subjective probability of bidder $i$. The expected utility of bidder $i$ for the target land under can be expressed as follows:

10 $$\begin{array}{c}E\left({x}_{i}\right)=\omega \left(\mathrm{G}\left({\psi }^{-1}\left({x}_{i}\right)\right)\right)\left({v}_{i}-{x}_{i}\right)\end{array}$$

In Eq. (10), $\left({v}_{i}-{x}_{i}\right)$ represents the bidder's utility of winning the target land.

##### **Proposition 3**

*Under assumption of bounded rationality II, the optimal bid strategy for a bidder with subjective decision probability is as follows.*

11 $$\begin{array}{c}{{x}_{i}}^{*}=\psi \left({v}_{i}\right)=\frac{1}{w\left(v\right)}{\int }_{\underline{v}}^{{v}_{i}}\xi tg\left(t\right){\left(w\left(t\right)\right)}^{2}Z\left(t\right)dt, {x}_{i}^{*}\epsilon \left[\underline{v},\overline{v }\right]\end{array}$$

$w\left(k\right)$ *denotes the twisted function of subjective probability* Eq. (9), $G\left(k\right)=F(k)^(n-1)$, $G\left(k\right)\in \left[\mathrm{0,1}\right]$. $Z\left(t\right)=\frac{{(1-G\left(t\right))}^{\xi -1}}{{G\left(t\right)}^{\xi +1}}$ *is defined for brevity.*

*The proof is in* Appendix A.

## Discussion

### Optimal bid strategy for bidders under the assumption of full rationality

#### **Corollary 1**

*The "hidden degree" of fully rational bidders' bids for the land is* ${\int }_{\underline{v}}^{{v}_{i}}{\left(\frac{F\left(t\right)}{F\left({v}_{i}\right)}\right)}^{n-1}dt$. *The more bidders there are in the same auction, the smaller the "hidden degree"; in short, competition will reduce the winning bidder's return space and increase the grantor's payoff.*

#### *Proof*

Under the assumption of full rationality, the optimal offer strategy of bidder *i* for the target land is ${v}_{i}-{\int }_{\underline{v}}^{{v}_{i}}{\left(\frac{F\left(t\right)}{F\left({v}_{i}\right)}\right)}^{n-1}dt$; then the bidder $i$’s surplus is ${\int }_{\underline{v}}^{{v}_{i}}{\left(\frac{F\left(t\right)}{F\left({v}_{i}\right)}\right)}^{n-1}dt$, and $\frac{\partial \phi }{\partial {v}_{i}}={F\left({v}_{i}\right)}^{1-2n}\left({v}_{i}{F\left({v}_{i}\right)}^{n}-F\left({v}_{i}\right){\int }_{0}^{{v}_{i}}t{f\left(t\right)}^{-1+n}dt\right){f\left({v}_{i}\right)}^{-1+n}\ge 0$ if and only if ${v}_{i}=0$ when $\frac{\partial \phi }{\partial {v}_{i}}=0$. Therefore, bidder $i$'s bidding strategy is a non-decreasing function concerning of his value, and $\frac{F\left(t\right)}{F\left({v}_{i}\right)}<1$, so that ${\left(\frac{F\left(t\right)}{F\left({v}_{i}\right)}\right)}^{n-1}$ is a decreasing function of $n$. As the number of bidders increases, the "hidden degree" of perfectly rational bidders decreases, and the surplus decreases.

#### **Corollary 2**

*If all bidders follow a perfectly rational best-bid strategy, only the bidder with the highest value wins the target land.*

#### *Proof*

It follows from Corollary 1 that the optimal bidding strategy for fully rational bidders is a non-decreasing function with respect to value, i.e., the bidder with the largest value will bid the highest, and the bidder with the highest value wins the auction according to the first-price sealed auction rule.

From Corollaries 1 and 2, it is clear that under the assumption of full rationality, the profit margin of bidders will continue decreasing as the degree of competition increases, and only the bidder with the highest value will win the target land. From the perspective of the grantor, the competition mechanism raises the revenue from the sale, which is the result the land grantor desires. In the past the residential market in real estate was in short supply^6^ Under favorable policy, developers have a sufficient profit margin and their value mainly depends on their development and operational efficiency. That is, the bidding decision under the assumption of full rationality is feasible in a period of rapid growth in the real estate market. However, to increase the possibility of winning, bidders can only increase their bids, but the profit margin is constantly forced down, and when the profit margin is not guaranteed, bidders will choose to leave the market. One of the implications of public auctions is that every bidder has a chance to win the land, which is a limitation of the assumption of full rationality. In the transition and upgrading period of the real estate market, policy control fluctuates, developers go bankrupt, land auctions and other phenomena occur frequently, and the value of developers is affected by various factors, and is no longer based on their efficiency.

### Optimal bid strategy for bidders under the assumption of bounded rationality

This study analyzes two different boundedly rational behaviors of bidders in China's open-bid auctions (i.e., auction and listing) and sealed-bid auctions (i.e., bid invitation). The Bayesian-Nash equilibrium in the two models is solved by establishing the RUM with distorted utility and the SDPM with distorted winning probability. The bidders' optimal bidding strategies in the two bounded rationality models constitute Propositions 2 and 3. This part will be discussed based on Propositions 2 and 3.

#### **Corollary 3**

*The bidding decisions in land auctions are significantly influenced by the economic environment. When the industry benchmark rate of return is higher, the bidder’s optimal bid price will be lower, and conversely, when the industry benchmark rate of return is lower, the bidder’s optimal bid price will be higher, given that bidders' value of the target land remains unchanged.*

#### *Proof*

From Proposition 2, it follows that the optimal bidding strategy for a bidder under the utility irrationality assumption is $\varphi \left({v}_{i}\right)=\frac{1}{\left(1+\lambda \right)\alpha }{\int }_{\underline{v}}^{{v}_{i}}t{\left[{F\left({v}_{i}\right)}^{-\frac{1}{\alpha }}{F\left(t\right)}^{-1+\frac{1}{\alpha }}f\left(t\right)\right]}^{n-1}dt$,$\frac{\partial \varphi \left({v}_{i}\right)}{\partial \lambda }=-\frac{1}{{\left(1+\lambda \right)}^{2}\alpha }{\int }_{\underline{v}}^{{v}_{i}}{t\left[{F\left[t\right]}^{-1+\frac{1}{\alpha }}{F\left[{v}_{i}\right]}^{-1/\alpha }{F}{\prime}\left[t\right]\right]}^{-1+n}dt<0$, which means that the bidder's bidding strategy is a decreasing function of $\lambda$.

Corollary 3 suggests that industry conditions (boom/bust) affect the attitudes that investors hold about future returns and thus influence their investment behavior (e.g., the land auction bids in this paper). That is, when the industry is booming, there are abundant investment opportunities to choose from in the market, and investors will not be obsessed with winning or losing a single auction; even if they fail to obtain the auctioned land parcel at one time, investors can turn to other projects for development and operation, which is manifested by their being willing to pay a lower price in land auctions. When the industry is in the doldrums, the overall market is capital-constrained, investment opportunities shrink, and investors face the risk of losing their projects without gaining revenue, so they are willing to pay higher prices in auctions to increase their chances of winning.

#### **Corollary 4**

*In bounded rationality assumptions I, under the same conditions, the greater the bidder's revenue preference for the target land (the greater the risk preference), the lower the bidder's optimal bid will be.*

#### *Proof*

The optimal bidding strategy of a bidder under bounded rationality assumptions I is $\varphi \left({v}_{i}\right)={G\left({v}_{i}\right)}^{-1/\alpha}{\int }_{\underline{v}}^{{v}_{i}}\frac{{tG\left(t\right)}^{-1+\frac{1}{\alpha }}g\left(t\right)}{\left(1+\lambda \right)\alpha }dt$ , the first-order derivative on the bidder's revenue attitude is that $\frac{\partial \varphi }{\partial \alpha }=\frac{{\int }_{\underline{v}}^{{v}_{i}}-t{G\left(t\right)}^{\frac{1}{\alpha }-1}(\alpha +In(G\left(t\right)))g(t)dt+In(G(v)){\int }_{\underline{v}}^{v}t{G\left(t\right)}^{-1+\frac{1}{\alpha }}g(t)dt}{(1+\lambda ){\alpha }^{3}{G({v}_{i})}^{-1/\alpha}}$, and since $0\le G({v}_{i})\le 1$, $In(G\left(t\right))<0$, and since the previous assumption $g(t)>0$, ${\int }_{\underline{v}}^{v}t{G\left(t\right)}^{-1+\frac{1}{\alpha }}g(t)dt>0$. Since $0<\alpha <1$, ${\int }_{\underline{v}}^{{v}_{i}}-t{G\left(t\right)}^{\frac{1}{\alpha }-1}(\alpha +In(G\left(t\right)))g(t)dt<0$; therefore $\frac{\partial \varphi }{\partial \alpha }<0$ holds constant. Simulated results are shown in Fig. 5b.

Corollary 4 shows that the bidder's revenue attitude toward the target land influences the bidding decision, which is different from the traditional value of land that influences the bidder's bid price but is influenced by the bidder's revenue preference for the target land. The practical implication is that when the bidder's value is certain, the degree of the bidder's risk preference will influence his bid; for example, if a developer has not been awarded land in previous centralized land auctions, then in the final land auction, the bidder’ eagerness to win the target land will weaken his revenue attitude; otherwise, he will face the next year of project development, so the bidder will increase his bid. This is an explanation of the bidding decision considering the psychological situation.

#### **Corollary 5**

*Bids for cost-advantaged developers increase as the decision-weight attitude factor increases, and bids for cost-disadvantaged developers decrease as the decision-weight attitude factor increases, but there is little change for cost-disadvantaged developers. In short, the decision-weight attitude causes indifferent bids from cost-disadvantaged bidders and increased competition from cost-advantaged developers.*

#### *Proof*

From Proposition 3, the optimal bidding strategy for a bidder under bounded rationality assumption II is equivalent to $\psi \left({v}_{i}\right)=\left(1+{\left(1-G\left({v}_{i}\right)\right)}^{\xi }{G\left({v}_{i}\right)}^{-\xi }\right){\int }_{\underline{v}}^{{v}_{i}}\frac{\xi t{\left(-\left(\left(-1+G\left(t\right)\right)G\left(t\right)\right)\right)}^{-1+\xi }g\left(t\right)}{{\left({\left(1-G\left(t\right)\right)}^{\xi }+{G\left(t\right)}^{\xi }\right)}^{2}}dt, G({v}_{i})$ being equal to ${F\left({v}_{i}\right)}^{n-1}$ , and $\frac{\partial \psi \left({v}_{i}\right)}{\partial \xi }={G\left({v}_{i}\right)}^{-\xi }(2ArcTanh\left[1-2G\left({v}_{i}\right)\right]{\left(1-G\left({v}_{i}\right)\right)}^{\xi }{\int }_{0}^{v}\frac{t\xi {\left(-\left(\left(-1+G\left({v}_{i}\right)\right)G\left({v}_{i}\right)\right)\right)}^{-1+\xi }g\left({v}_{i}\right)}{{\left({\left(1-G\left({v}_{i}\right)\right)}^{\xi }+{G\left({v}_{i}\right)}^{\xi }\right)}^{2}}dt+\left({\left(1-G\left({v}_{i}\right)\right)}^{\xi }+{G\left({v}_{i}\right)}^{\xi }\right){\int }_{0}^{v}-\frac{t{\left(-\left(\left(-1+G(t)\right)G(t)\right)\right)}^{-1+\xi }\left(\left(-1+2\xi \mathrm{ArcTanh}\left[1-2G(t)\right]\right){\left(1-G(t)\right)}^{\xi }-\left(1+2\xi \mathrm{ArcTanh}\left[1-2G(t)\right]\right){G(t)}^{\xi }\right)g(t)}{{\left({\left(1-G(t)\right)}^{\xi }+{G(t)}^{\xi }\right)}^{3}}dt)$. Due to the probability of winning bid satisfies $0\le G\left({v}_{i}\right)<1$, ${\int }_{0}^{v}\frac{t\xi {\left(-\right)}^{-1+\xi }g\left({v}_{i}\right)}{{\left({\left(1-G\left({v}_{i}\right)\right)}^{\xi }+{G\left({v}_{i}\right)}^{\xi }\right)}^{2}}dt$ is constantly greater than 0, let $G\left({v}_{0}\right)=0.5$, when ${v}_{i}>{v}_{0}$ when $2ArcTanh\left[1-2G\left({v}_{i}\right)\right]<0$, then $\frac{\partial \psi \left({v}_{i}\right)}{\partial \xi }<0$, and vice versa, when ${v}_{i}<{v}_{0}$ when $\frac{\partial \psi \left({v}_{i}\right)}{\partial \xi }>0$. That is, for the dominant developer $({v}_{i}>{v}_{0})$ bids increase as the decision weight attitude coefficient increases, and bids for the weaker developers $({v}_{i}<{v}_{0})$ decrease as the decision weight attitude factor increases. As shown in Fig. 5c in “Numerical simulation” section.

Corollary 5 suggests that a decision-weighted attitude would render the bids of cost-disadvantaged bidders indifferent, while competition from cost-advantaged developers intensifies. Such an outcome will lead to the consolidation and optimization of property developers, screening out disadvantaged developers who entered the property industry due to high profits a few years ago, in line with the laws of the market.

#### **Corollary 6**

*Regardless of the extent to which competition increases, in land auctions, bidders not only pay no more than their values but also retain profit margins, depending on industry benchmark yields and bidders' preference for yield.*

#### *Proof*

**Figure 3:**
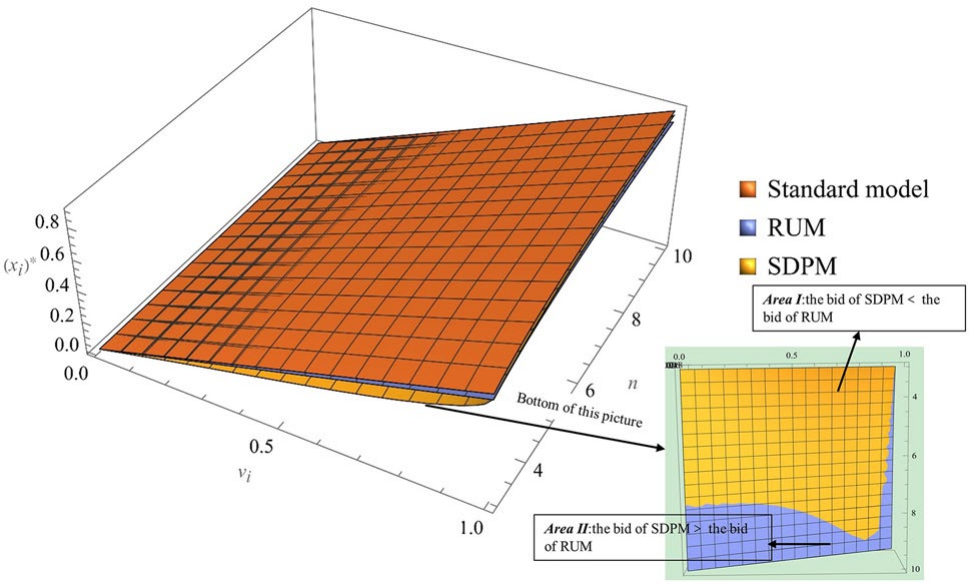
The shape of bidding strategy under different value and competition.

The optimal bidding strategy of bidders in the RUM is $\varphi \left({v}_{i}\right)=\frac{1}{(1+\lambda )\alpha }{\int }_{\underline{v}}^{{v}_{i}}t{[{F\left({v}_{i}\right)}^{-\frac{1}{\alpha }}{F\left(t\right)}^{-1+\frac{1}{\alpha }}f\left(t\right)]}^{n-1}dt=\frac{1}{(1+\lambda )\alpha }{\int }_{\underline{v}}^{{v}_{i}}t{[{(\frac{F\left(t\right)}{F\left({v}_{i}\right)})}^{\frac{1}{\alpha }}\frac{f\left(t\right)}{F\left(t\right)}]}^{n-1}dt$, due to $0<\alpha <1$, $\frac{F\left(t\right)}{F\left({v}_{i}\right)}<1$, $\frac{f\left(t\right)}{F\left(t\right)}>1$, so when $n\to \infty$ when ${[{(\frac{F\left(t\right)}{F\left({v}_{i}\right)})}^{\frac{1}{\alpha }}\frac{f\left(t\right)}{F\left(t\right)}]}^{n-1}\ne 0$ and $\varphi \left({v}_{i}\right)\ne {v}_{i}$, due to $\varphi \left({v}_{i}\right)\le {v}_{i}$, so there is a profit margin for the bidders. Reference can be made to Fig. 3 to visualize the results for comparison and verification.

Corollary 6 suggests that bidders will consider their surplus space no matter how much competition increases. In reality, social development also requires the protection of developers' basic living space. This is also the land auction mechanism designers need to consider in advance.

## Numerical simulation

In this section, the standard model, RUM, and SDPM are visualized using computer numerical simulations to more intuitively describe the relevant inferences. To facilitate the calculation, similar to established studies^18^, it is assumed here that the bidders' value of the land ${v}_{i}$ obeys a uniform distribution over the interval $\left[\mathrm{0,1}\right]$ on a uniform distribution, and for the sake of generality, the $\lambda$ the average net interest rate of 9.8% from the Research Report on China's Top 100 Real Estate Enterprises published by the CMI Institute is chosen.

First, a set of random numbers is generated by numerical simulation as the value of different bidders in the same auction ${v}_{i}$ and the optimal bidding strategies of different bidders are calculated by the results of the model ${{x}_{i}}^{*}$ This will be the source of the data in this section. Table 3 reports the simulated results for the standard model, the RUM, and the SPDM under the condition $n=9$ , $\alpha =0.88$ and $\xi =0.61$^7^.

Table 3 shows different bids of 9 bidders solved by the standard model, RUM and SDPM, given the simulated value, ${v}_{i}$. It can be seen that the bids under the RUM and SDPM (i.e., bounded rationality model) are lower than the bids under the standard model, This indicates that human psychological and environmental factors have significant effects on bidders' decisions, that subjective distortions in bidders' utilities and subjective distortions in decision weights cause bidders' bidding strategies to deviate from the standard model, and that boundedly rational bidders bid less than fully rational bidders.

**Table 3:** A set of random simulation data obtained when $n=9$, $\alpha =0.88$, $\xi =0.61$.

^Bidder*i*^ _*Bid*_	1	2	3	4	5	6	7	8	9
${v}_{i}$	0.859	0.929	0.425	0.568	0.620	0.221	0.171	0.023	0.849
Standard model	0.764	0.826	0.378	0.505	0.551	0.196	0.152	0.021	0.754
RUM	0.705	0.763	0.349	0.466	0.509	0.181	0.140	0.019	0.696
SDPM	0.700	0.760	0.353	0.469	0.511	0.183	0.142	0.019	0.691

We are also interested in the impact of competition intensity on bidding decisions and land grantors in auctions. From the bidders' perspective, we focus on the change in the strategies of bidders with the same value under different competitive intensities; from the land grantor's perspective, we focus on the highest bid, i.e., the sales price, in the same auction under different competitive intensities. Using the number of bidders participating in the auction $n$, we simulate the bidding strategies of different bidders and their highest bids.

**Figure 4:**
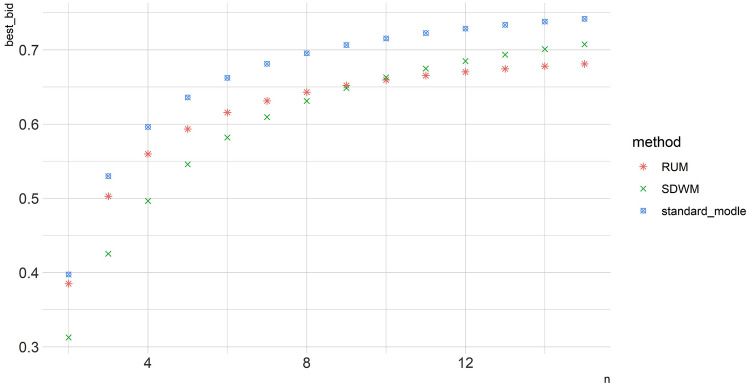
Simulation bid price under different competition.

As shown in Fig. 3, the bidders' bidding strategies increase with the intensity of competition in all three models, but the deviation between boundedly rational bidding and fully rational bidding increases as developer competition intensifies. In addition, the optimal bids of the bidders in the RUM and the SDPM are lower than those in the standard model, the SDPM bids are lower than the RUM bids in Region I, and the RUM bids are lower than the SDPM bids in Region II. As shown in Figs. 3 and 4, increased competition increases the surplus of the land grantor; however, when competition increases enough, the standard model bids tend toward $\overline{v}$ and the RUM will always retain surplus space.

Finally, the influence of $\lambda$, $\alpha$ and $\xi$ on bidding strategies is simulated, considering different benchmark yields in the real estate industry in different countries and regions (i.e., $\lambda$), different attitudes toward earnings (i.e., $\alpha$) and different attitudes of bidders' decision weights (i.e., $\xi$).

**Figure 5:**
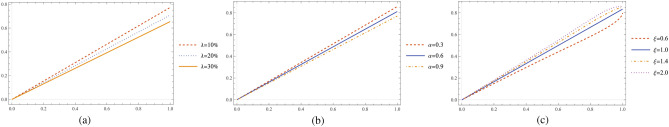
The simulation λ, α, ξ impact on bidders' bidding strategies.

As shown in Fig. 5a, the bidders' bid decreases with the real estate industry benchmark rate $\lambda$. This suggests that, ceteris paribus, when the economy is booming, the bidder's bidding strategy will be lower and the bidder expects to make a larger profit. This finding is similar to Wang et al.^15^. As shown in Fig. 5b, the bidder's bid decreases with revenue attitude *α*. These results have been confirmed in other industries (e.g., the electricity market^33^). Figure 5c shows that the optimal bid strategy in the SDPM is an "inverse S" curve, and the cost-disadvantaged bidder is similar to the standard model ($\xi =1$). $\xi = 0.6$ is more consistent with behavioral economics research.

As shown in Fig. 5, when ξ = 0.6, the cost-advantaged developer bid lower (compared to ξ = 1). Cost-advantaged developers might have a special relationship with the owner, a brand premium and a qualification advantage, which gives them more confidence to bid lower prices^34^. In other words, in sealed-bid auctions, the "hiding degree" of cost-advantaged developers is greater. From the experience of developed countries^35^, this result will lead to size effects in sealed-bid auctions. However, the bids in open-bid auctions are more complex and affected by market conditions and developers’ risk preferences.

## Conclusion

When bidding for land, real estate developers in China tend to show different boundedly rational behaviors in different auction formats, which leads to deviation from the bidding strategy under full rationality. As China’s real estate industry transforms, this deviation is amplified and cannot be ignored. To address this, this study establishes two bounded rationality models to depict developers’ subjective distortions of utility and winning probability in open-bid and sealed-bid auctions. On this basis, the deviation between boundedly rational bidding and fully rational bidding in land auctions is characterized and interpreted, and the applicable conditions of the two models are analyzed, which provides better decision support for developers and a more realistic basis for land auction mechanism design. The main conclusions are as follows.

Firstly, the deviation between boundedly rational bidding and fully rational bidding increases with competition. Specifically, boundedly rational bidding grows more slowly, which means that developers will not raise their bids as much to increase their winning probability at the cost of sacrificing their profit margin. On the contrary, they are more willing to retain a certain surplus, which is exactly denoted by the deviation. Secondly, compared with cost-advantaged developers, the boundedly rational bidding of cost-disadvantaged developers deviates less from the standard model, indicating that they have less ability to retain profits. In other words, cost-disadvantaged developers have to sacrifice more profit to win the auction. In contrast, cost-advantageous developers can retain more profits in land auction bidding under bounded rationality; that is, their “hiding degree” from fully rational bidding is higher. More precisely, in sealed-bid auctions, the “hiding degree” of cost-advantaged developers is greater, showing size effects, which is consistent with the experience developed countries in Europe. However, in open-bid auctions, their bids are more complex and affected by market conditions and developers’ risk preferences.

There are some limitations. Firstly, this study is based on the first-price sealed auction mechanism. More auction formats can be explored in further studies. Secondly, in terms of bounded rationality, this study only considers the subjective distortion of utility and winning probability. Further studies can incorporate more kinds of boundedly rational behaviors, e.g., expected regret. Moreover, this study verifies the model parameters only by numerical simulations. Future studies may test the validity of the model through behavioral experiments and empirical studies.

### Supplementary Information

Supplementary Information.

## Data Availability

The datasets used and/or analysed during the current study available from the corresponding author on reasonable request.
