# Supplementary material for: Boundedly rational bidding decision for land auctions during the transformation of real estate markets
Source: Sci Rep. 2023 Sep 9;13:14889. doi: 10.1038/s41598-023-41993-7 (PMC10492829; doi:10.1038/s41598-023-41993-7)
Supplement: Supplementary file 1 — Supplementary Information. [file 41598_2023_41993_MOESM1_ESM.docx]

# Appendix A

**Proposition 1:** Under the assumption of full rationality bidders$i$ 's optimal bid offer:

$$\begin{aligned} x_{i}^{*}=\phi\boldsymbol{(}v_{i})=v_{i}-\int_{\underline{v}}^{v_{i}} \left( \frac{F\left( t \right)}{F\left( v_{i} \right)} \right)^{n-1}dt, x_{i}^{*}\epsilon\left[ \underline{v},\bar{v} \right]\# \end{aligned}$$

**Proof:**

Bidder i's expected return on the target land is

$$\begin{aligned} E(x_{i})=G\left( \phi^{\boldsymbol{-1}}\left( x_{i} \right) \right)\times\pi_{i}=G\left( \phi^{-1}\left( x_{i} \right) \right)\times\left( v_{i}-x_{i} \right)\#\left( A-1 \right) \end{aligned}$$

$G\left( \phi^{\boldsymbol{-1}}\left( x_{i} \right) \right)$ means $Pr[x_{i}>\max_{j\neq i} x_{j}]$. $v_{i}-x_{i}$ is the return of winning the bid. To maximize the expected utility of bidders $i$, the first-order condition of the utility function is obtained:

$$\begin{aligned} \frac{\partial\left( E^{j=1}\left( x_{i} \right) \right)}{\partial\left( x_{i} \right)}=\frac{g\left( \phi^{-1}\left( x_{i} \right) \right)}{\phi^{'}\left( \phi^{-1}\left( x_{i} \right) \right)}\left( v_{i}-x_{i} \right)-G\left( \phi^{-1}\left( x_{i} \right) \right)=0\#\left( A-2 \right) \end{aligned}$$

In equation (A-2) $g$ =$G^{'}$ denotes the probability density function of bidders' bids. In the symmetric equilibrium $x_{i}=\phi\left( v_{i} \right)$, the equivalent differential equation (A-3) can be obtained:

$$\begin{aligned} \frac{d}{d\left( v_{i} \right)}\left[ G\left( v_{i} \right)\phi\left( v_{i} \right) \right]=(v_{i})g\left( v_{i} \right)\#\left( A-3 \right) \end{aligned}$$

There are boundary conditions where $\phi\left( 0 \right)=0$, then the general solution of the differential equation is:

$$\begin{aligned} \phi\left( v_{i} \right)=\frac{1}{G\left( v_{i} \right)}\int_{\underline{v}}^{v_{i}} tg\left( t \right)dt=E\left\{ T_{1} | T_{1}<v_{i} \right\}\#\left( A-4 \right) \end{aligned}$$

In equation (A-4)$T_{1}\equiv T_{1}^{\left( n-1 \right)}$ , denotes the highest valuation among the remaining $n-1$ bidders. The above equation is its sufficiency, and its necessity is proved as follows: if the bidders' valuations are$v_{i}$ of the offer $x_{i}=\phi\left( z \right)$ **,** then the bidder $i$ 's expected utility is:

$$\begin{aligned} E\left( x_{i} \right)=G\left( z \right)\left[ v_{i}-\phi\left( z \right) \right]=G\left( z \right)\left( v_{i}-E\left\{ T_{1} | T_{1}<z \right\} \right) \\ =v_{i}G\left( z \right)-\int_{\underline{v}}^{\boldsymbol{z}} tg\left( t \right)dt=\left( v_{i}-z \right)G\left( z \right)+\int_{\underline{v}}^{z} G\left( t \right)dt\#\left( A-5 \right) \end{aligned}$$

At this point, either $z\boldsymbol{\geq}v_{i}$ or $z<v_{i}$ the expected return is greater than or equal to 0, then the original equation holds.

This means that once all the other bidders have adopted the offer strategy $\phi$ , bidder $i$ has and only obtains the payoff through bidding strategy $\phi\left( v_{i} \right)$, i.e., equation (A-4) is a symmetric equilibrium offer strategy. Since $G\left( x \right)E\left( X | X<x \right)=\int_{\underline{v}}^{x} tg\left( t \right)dt=xG\left( x \right)-\int_{\underline{v}}^{x} G\left( t \right)dt$ , This is because the expectation of X is $E[X] =\int_{0}^{\omega} xf\left( x \right)dx$ if X obeys the distribution F. And if $\gamma: [0,\omega\mathbb{]\to R}$ is any function, then, similarly, the expectation of $\gamma(X)$ can be defined to be $E\left[ \gamma\left( x \right) \right]=\int_{0}^{\omega} \gamma\left( x \right)f\left( x \right)dx$. Given $X<x$, the conditional expectation of $X$ is $E\left( X | X<x \right)=\frac{1}{F(x)}\int_{0}^{x} tf\left( t \right)dt$. Obtaining the above $G\left( x \right)E\left( X | X<x \right)=\int_{\underline{v}}^{x} tg\left( t \right)dt=xG\left( x \right)-\int_{\underline{v}}^{x} G\left( t \right)dt$*.* Since $\frac{G\left( t \right)}{G\left( v_{i} \right)}=\left[ \frac{F\left( t \right)}{F\left( v_{i} \right)} \right]^{n-1}$, the equilibrium bid of bidder $i$ can be written

$$\phi\left( v_{i} \right)=v_{i}-\int_{\underline{v}}^{v_{i}} \frac{G\left( t \right)}{G\left( v_{i} \right)}dt=v_{i}-\int_{\underline{v}}^{v_{i}} \left[ \frac{F\left( t \right)}{F\left( v_{i} \right)} \right]^{n-1}dt$$

And，$\phi\left( v_{i} \right)\in\left[ \underline{v}, \overline{v}-\int_{\underline{v}}^{\overline{v}} \left[ \frac{F\left( t \right)}{F\left( \overline{v} \right)} \right]^{n-1}dt \right]$

**Proposition 2:** The optimal bid offer strategy for a bounded rational bidder for a utility irrationality is

$$\begin{aligned} {x_{i}}^{*}=\frac{1}{\left( 1+\lambda\right)\alpha}\int_{\underline{v}}^{v_{i}} \frac{{tG\left( t \right)}^{-1+\frac{1}{\alpha}}g\left( t \right)}{{G\left( v_{i} \right)}^{1/\alpha}}ⅆt, x_{i}^{*}\epsilon\left[ \underline{v},\bar{v} \right]\# \end{aligned}$$

**Proof:**

The expected utility function of bidder $i$ under the RUM is as follows.

$$\begin{aligned} E_{\left( + \right)}\left( x_{i} \right)=G\left( \varphi^{\boldsymbol{-1}}\left( x_{i} \right) \right)*\left( r_{0}-x_{i} \right)^{\alpha} \#\left( A-6 \right) \end{aligned}$$

$$\begin{aligned} E_{\left( - \right)}\left( x_{i} \right)=G\left( \varphi^{\mathbf{-1}}\left( x_{i} \right) \right)\left( {-\eta\left( x_{i}-r_{0} \right)}^{\beta} \right) \#\left( A-7 \right) \end{aligned}$$

$E_{\left( + \right)}\left( x_{i} \right)$ and $E_{\left( - \right)}\left( x_{i} \right)$ denote the subjective expected utility of the bidder compared to the expected gain and loss at the reference point, respectively. $G\left( \varphi^{\boldsymbol{-1}}\left( x_{i} \right) \right)$ is the bidder $i$’s objective probability of winning the bid. $\alpha$ and $\beta$ denote the coefficients of bidders' preferences for exceeding and underperforming peer returns, respectively. $\eta$ denotes the bidder's preference for risk aversion parameters. Take the first derivative of the above equation, the first-order derivative condition is obtained as:

$$\begin{aligned} \frac{\partial E_{\left( + \right)}\left( x_{i} \right)}{\partial x_{i}}=\left( r_{0}-x_{i} \right)^{\alpha}\left( -\frac{\alpha G\left( \varphi^{\boldsymbol{-1}}\left( x_{i} \right) \right)}{r_{0}-x_{i}}+\frac{g\left( \varphi^{\boldsymbol{-1}}\left( x_{i} \right) \right)}{\varphi^{\boldsymbol{'}}\left( \varphi^{\boldsymbol{-1}}\left( x_{i} \right) \right)} \right)=0 \#\left( A-8 \right) \end{aligned}$$

$$\begin{aligned} \frac{\partial E_{\left( - \right)}\left( x_{i} \right)}{\partial x_{i}}=\left( x_{i}-r_{0} \right)^{\beta}\eta(\frac{\beta G\left( \varphi^{\boldsymbol{-1}}\left( x_{i} \right) \right)}{r_{0}-x_{i}}-\frac{g\left( \varphi^{\boldsymbol{-1}}\left( x_{i} \right) \right)}{\varphi^{\boldsymbol{'}}\left( \varphi^{\boldsymbol{-1}}\left( x_{i} \right) \right)}=0 \#\left( A-9 \right) \end{aligned}$$

In the above equation $g$ =$G^{'}$ denotes the probability density function of bidders' bids, and $r_{0}=\frac{v_{i}}{1+\lambda}$ . Since in the symmetric equilibrium$\left( r_{0}-x_{i} \right)^{\alpha-1}>0$ and $\eta\left( x_{i}-r_{0} \right)^{\beta}>0$, $x_{i}=\varphi\left( v_{i} \right)$, then the differential equation can be obtained:

$$\begin{aligned} \alpha G\left( v_{i} \right)=\left( r_{0}-\varphi\left( v_{i} \right) \right)\frac{g\left( v_{i} \right)}{\varphi^{\boldsymbol{'}}\left( v_{i} \right)}\#\left( A-10 \right) \end{aligned}$$

$$\begin{aligned} \frac{\beta G\left( v_{i} \right)}{r_{0}-x_{i}}=\frac{g\left( v_{i} \right)}{\varphi^{\boldsymbol{'}}\left( v_{i} \right)}\#\left( A-11 \right) \end{aligned}$$

After the above derivation, it is found that $\eta$ has no influence on the bidder's bidding decision. In other words, the bidder will realize the existence of the loss state when he bids, and thus the bidder will give up bidding due to the risk aversion utility. Therefore, the two cases can be considered together, and only the different attitude coefficients of the bidders can be considered. At the same time, a bidder with a valuation of 0 will not submit an offer greater than 0 because he incurs a loss instead if he wins the auction. Therefore, we are bound to get $\varphi\left( 0 \right)=0$. Finally, we just need to find the complementary solution for the differential equation ( A-11).

Separating the variables for the differential equation (A-11) yields $d\varphi\left( v_{i} \right)=\frac{{(r}_{0}-x_{i})g(v_{i})}{\beta G(v_{i})}dv_{i}$. Integrating both sides of the equal sign after substituting $r_{0}=\frac{v_{i}}{1+\lambda}$ and $x_{i}=\varphi\left( v_{i} \right)$ yields, $\varphi\left( v_{i} \right)=c_{1}{G\left( v_{i} \right)}^{{-1}/\alpha}+{G\left( v_{i} \right)}^{{-1}/\alpha}\int_{\underline{v}}^{v_{i}} \frac{{tG\left( t \right)}^{-1+\frac{1}{\alpha}}g\left( t \right)}{\left( 1+\lambda\right)\alpha}ⅆt$*.* Finally, $\varphi\left( 0 \right)=0$, we can obtain that $c_{1}=0$.

The optimal bidding strategy for the bidder under the reference utility model is

$$\begin{aligned} \varphi\left( v_{i} \right)={G\left( v_{i} \right)}^{{-1}/\alpha}\int_{\underline{v}}^{v_{i}} \frac{{tG\left( t \right)}^{-1+\frac{1}{\alpha}}g\left( t \right)}{\left( 1+\lambda\right)\alpha}ⅆt\#\left( A-12 \right) \end{aligned}$$

In equation (A-12), the $G\left( v \right)$ denotes the distribution function of bidders' bids. Similar to the Standard Model, for all bids $G\left( v \right)={F(v)}^{n-1}$ . Then we obtain the optimal bid-offer strategy under the bounded rationality assumptions Ⅰ, where bidders behave irrationally for subjective evaluation of utility.

**Proposition 3**: Under the assumption of bounded rationality II, the optimal bid strategy for a bidder with Subjective decision probability for the land is $\begin{aligned} {x_{i}}^{*}=\psi\left( v_{i} \right)=\frac{1}{w\left( v \right)}\int_{\underline{v}}^{v_{i}} \xi tg\left( t \right)\left( \omega\left( t \right) \right)^{2}Z\left( t \right)dt, x_{i}^{*}\epsilon\left[ \underline{v},\bar{v} \right]\# \end{aligned}$

$\omega\left( k \right)$ denotes the twisted function of subjective probability Eq. (9), the $G\left( k \right)=F(k)^(n-1)$ , $G\left( k \right)\in\left[ 0,1 \right]$. $Z\left( t \right)=\frac{{(1-G\left( t \right))}^{\xi-1}}{{G\left( t \right)}^{\xi+1}}$ is defined for brevity.

**Proof:**

The expected utility of the bidder $i$ for the target land under can be expressed as follows:$\begin{aligned} E\left( x_{i} \right)=\frac{{G\left( \psi^{\mathbf{-1}}\left( x_{i} \right) \right)}^{\xi}}{{G\left( \psi^{\mathbf{-1}}\left( x_{i} \right) \right)}^{\xi}+\left( 1-G\left( \psi^{\mathbf{-1}}\left( x_{i} \right) \right) \right)^{\xi}}\left( v_{i}-x_{i} \right)\#\left( A-13 \right) \end{aligned}$

In formula (A-14) $G\left( \psi^{\mathbf{-1}}\left( x_{i} \right) \right)$ is the bidder $i$'s objective probability of winning the bid, similarly to before, $G=F^(n-1)$. $\xi$ denotes the bidder's decision weight attitude, and$\left( v_{i}-x_{i} \right)$ represents the bidder's utility of winning the target land. Bidder $i$ is seeking to maximize the expected utility of his subjective decisions, so the first-order derivative condition of equation (19) is:

$$\begin{aligned} \frac{\partial E^{j=2}\left( x_{i} \right)}{\partial x_{i}}={G\left( \psi^{-1}\left( x_{i} \right) \right)}^{-1+\xi}\frac{\left( -\left( \left( -1+G\left( \psi^{-1}\left( x_{i} \right) \right) \right){G\left( \psi^{-1}\left( x_{i} \right) \right)}^{1+\xi} \right)+\left( 1-G\left( \psi^{-1}\left( x_{i} \right) \right) \right)^{\xi}\left( G\left( \psi^{-1}\left( x_{i} \right) \right)-{G\left( \psi^{-1}\left( x_{i} \right) \right)}^{2}+\left( -v_{i}+x_{i} \right)\xi g \left( \psi^{-1}\left( x_{i} \right) \right) \right) \right)}{\psi^{'}\left( \psi^{-1}\left( x_{i} \right) \right)\left( \left( -1+G\left( \psi^{-1}\left( x_{i} \right) \right) \right)\left( \left( 1-G\left( \psi^{-1}\left( x_{i} \right) \right) \right)^{\xi}+{G\left( \psi^{-1}\left( x_{i} \right) \right)}^{\xi} \right)^{2} \right)}\#\left( A-14 \right) \end{aligned}$$

In the above equation $g$ =$G^{'}$ denotes the probability density function of bidders' bids, and $x_{i}=\psi\left( v_{i} \right)$ , then the differential equation (A-15) can be obtained:

$$\begin{aligned} \frac{\xi\left( 1-G\left( v_{i} \right) \right)^{-1+\xi}\left( v-\psi\left( v_{i} \right) \right)g\left( v_{i} \right)}{G\left( v_{i} \right)\psi^{'}\left( v_{i} \right)}=\left( 1-G\left( v_{i} \right) \right)^{\xi}+{G\left( v_{i} \right)}^{\xi}\#\left( A-15 \right) \end{aligned}$$

For the differential equation (A-15) separating the variables and integrating on both sides yields the complementary solution $\psi\left( v_{i} \right)=\left( 1+\left( 1-G\left( v_{i} \right) \right)^{\xi}{G\left( v_{i} \right)}^{-\xi} \right)(c_{1}+\int_{\underline{v}}^{v_{i}} \frac{\xi t\left( -\left( \left( -1+G\left( t \right) \right)G\left( t \right) \right) \right)^{-1+\xi}g\left( t \right)}{\left( \left( 1-G\left( t \right) \right)^{\xi}+{G\left( t \right)}^{\xi} \right)^{2}}ⅆt)$. At the same time, a bidder with a valuation of 0 will not submit an offer greater than 0 because he incurs a loss instead if he wins the auction. Therefore, we are bound to get $\psi\left( 0 \right)=0$. Therefore, the equilibrium solution for bidders under the subjective decision probability model is

$$\begin{aligned} \psi\left( v_{i} \right)=\left( 1+\left( 1-G\left( v_{i} \right) \right)^{\xi}{G\left( v_{i} \right)}^{-\xi} \right)\int_{\underline{v}}^{v_{i}} \frac{\xi t\left( -\left( \left( -1+G\left( t \right) \right)G\left( t \right) \right) \right)^{-1+\xi}g\left( t \right)}{\left( \left( 1-G\left( t \right) \right)^{\xi}+{G\left( t \right)}^{\xi} \right)^{2}}ⅆt\#\left（ A-16 \right） \end{aligned}$$

For simplicity and ease of observation, the equation can be substituted as

$$\begin{aligned} \psi\left( v_{i} \right)=\frac{1}{\omega\left( v_{i} \right)}\int_{\underline{v}}^{v_{i}} \xi tg\left( t \right)\left( \omega\left( t \right) \right)^{2}Z\left( t \right)dt\#\left( A-17 \right) \end{aligned}$$

$\omega\left( k \right) denotes the twisted function of subjective probability Eq. (9)$, the $G\left( k \right)=F(k)^(n-1)$ , $G\left( k \right)\in\left[ 0,1 \right]$ , and$Z\left( t \right)=\frac{{(1-G\left( t \right))}^{\xi-1}}{{G\left( t \right)}^{\xi+1}}$ .
